# Repair abilities of mouse autologous adipose-derived stem cells and ShakeGel™3D complex local injection with intrauterine adhesion by BMP7-Smad5 signaling pathway activation

**DOI:** 10.1186/s13287-021-02258-0

**Published:** 2021-03-18

**Authors:** Yun-xia Zhao, Shao-rong Chen, Qiao-yi Huang, Wei-can Chen, Tian Xia, Yan-chuan Shi, Hong-zhi Gao, Qi-yang Shi, Shu Lin

**Affiliations:** 1 Department of Gynaecology and Obstetrics, The Second Affiliated Hospital of Fujian Medical University, No.34 North Zhongshan Road, Quanzhou, 362000 Fujian Province China; 2 Department of Anaesthesiology, the Second Affiliated Hospital, Fujian Medical University, Quanzhou, China; 3 School of Pharmaceutical Sciences, Xiamen University, Xiamen, 361102 Fujian province China; 4 Diabetes and Metabolism Division, Garvan Institute of Medical Research, 384 Victoria Street, Darlinghurst, Sydney, NSW 2010 Australia; 5 Faculty of Medicine, St Vincent’s Clinical School, Univeristy of New South Wales, Sydeny, New South Wales 2052 Australia; 6 Clinical Center for Molecular Diagnosis and Therapy, the Second Affiliated Hospital of Fujian Medical University, Quanzhou, Fujian China; 7 Centre of Neurological and Metabolic Research, the Second Affiliated Hospital of Fujian Medical University, No.34 North Zhongshan Road, Quanzhou, 362000 Fujian Province China

**Keywords:** Autologous adipose-derived stem cells, ShakeGel™3D, Intrauterine adhesion, BMP7-Smad5 signaling pathway

## Abstract

**Background:**

The objective was to explore the therapeutic effect of autologous adipose-derived stem cells (ADSCs) combined with ShakeGel™3D transplantation to activate the BMP7-Smad5 signaling pathway to treat intrauterine adhesions (IUA).

**Methods:**

Autologous ADSCs were isolated and then merged with ShakeGel™3D. The IUA model was established by mechanical injury. The third generation of autologous ADSCs was injected directly into the uterus in combination with ShakeGel™3D. After 7 days of treatment, endometrial morphology, number of endometrial glands, endometrial fibrosis area, and fibrosis biomarker analysis by RT-PCR and IHC were examined. BMP7 and phosphorylation of Smad5 were also detected, and the recovery of infertility function in treated mice was evaluated.

**Results:**

Fluorescence-activated cell sorting (FACS) showed that autologous ADSCs expressed CD105 (99.1%), CD29 (99.6%), and CD73 (98.9%). Autologous ADSCs could still maintain a good growth state in ShakeGel™3D. Histological examination revealed that the number of endometrial glands increased significantly, and the area of fibrosis decreased. At the same time, the expression of BMP7 and Smad5 in the ADSCs + Gel group was significantly upregulated, and the final reproductive function of this group was partly recovered.

**Conclusions:**

Autologous ADSCs can be used in combination with ShakeGel™3D to maintain functionality and create a viable three-dimensional growth environment. The combined transplantation of autologous ADSCs and ShakeGel™3D promotes the recovery of damaged endometrial tissue by increasing BMP7-Smad5 signal transduction, resulting in endometrium thickening, increased number of glands, and decreased fibrosis, leading to restoration of partial fertility.

**Supplementary Information:**

The online version contains supplementary material available at 10.1186/s13287-021-02258-0.

## Background

Intrauterine adhesions (IUA), the most common cause of uterine infertility, partially or completely block the uterine cavity and/or cervical canal and are caused by endometrial basal layer injury. There are several hypotheses about the pathogenesis of IUA, including the fibrosis hyperplasia theory, the neural reflex theory, abnormal differentiation of stem cells, changes in the uterine microenvironment and fibrosis, abnormal regulation of signaling pathways, and inflammatory response caused by adherent fibroblasts. Since the pathological features of IUA are endometrial fibrosis, the fibrosis hyperplasia theory is the most widely studied hypothesis. As a serious complication of uterine surgery, IUA can cause a plethora of female reproductive system health problems, such as menstrual abnormalities, recurrent abortion, periodic abdominal pain, infertility, and pregnancy-related complications. Therefore, the goal of IUA therapy is to reconstruct the normal uterine cavity and restore uterine function. The traditional treatment for this disease is hysteroscopic resection of transcervical adhesion, combined with various adjuvant therapies. However, some shortcomings and deficiencies of these anti-adhesion strategies, such as resistance to secondary surgery, limited isolation area, induction of intrauterine inflammatory response, and difficulty in endometrial regeneration, have forced researchers to continue to explore new treatment options.

Mesenchymal stem cells (MSCs) have the ability of multipotent differentiation, they can promote tissue repair, and they are characterized by low immunoreactivity and high immunosuppression. They are a suitable source of stem cells for the treatment of intrauterine adhesions. However, the safety of allogeneic MSCs regeneration therapy remains controversial. Compared with autologous MSCs, allogeneic MSCs have a higher risk of initiating an immune response [1], higher morbidity [2], and secondary cancers that may occur after allogeneic stem cell transplantation [3]. According to recent experiments and reports, autologous MSCs seem to be more suitable for the treatment of diseases [4–7]. Adipose-derived stem cells (ADSCs), a type of MSCs, have shown promise in regenerative medicine. In preclinical animal models, autologous ADSCs are used to repair articular cartilage defects [8], enhance the recovery of erectile function after cavernous nerve injury [9], and repair Achilles tendon defects [10]. Furthermore, autologous ADSCs have been used in the treatment of Crohn’s disease anal fistula [11], systemic sclerosis [12], and tracheomediastinal fistula in humans [13]. In addition, we reviewed the application of MSCs in female reproductive diseases [14]. Currently, the primary mechanisms of MSCs therapy have not been fully elucidated. The main mechanisms include differentiation into targeted cells, immunomodulatory interactions with multiple immune cells, homing and engraftment at targeted injury sites, and paracrine effects of various factors, among which the paracrine effect is the most likely mode of action.

In order to improve the local persistence and utilization rate of MSCs in the endometrium, scientists have used materials such as collagen scaffolds [15–17], hydrogel scaffolds [18], and platelet-rich plasma [19] to loaded with MSCs to restore the structure and function of the endometrium. Our experiments used a material with a three-dimensional polymeric network, a bioactive hydrogel mimicking the microenvironment and microstructure of tissues, supporting 3D tissue-like growth in vitro and in vivo [20]. ShakeGel™3D can also improve the proliferation or differentiation of stem cells.

Bone morphogenetic protein 7 (BMP7) is a member of the transforming growth factor (TGF) superfamily and a natural negative regulator of the TGF/Smad signaling pathway. Recently, a number of studies have found that BMP7 can inhibit the progression of cardiac fibrosis [21], glomerular and interstitial fibrosis in mouse models of chronic renal disease [22, 23], pulmonary fibrosis [24], and IUA [25]. Furthermore, experiments have shown that BMP7 may suppress TGF-β1, which mediates IUA fibrosis by stimulating Smad and downstream regulatory pathways [26–30], inducing the process by regulating Smad5 to repair tissues [25].

In this study, we evaluated the therapeutic effects of a complex of mouse autologous ADSCs loaded on ShakeGel™3D in an IUA mouse model by activating the BMP7-Smad5 signaling pathway, which increased local perseverance and stem cells activity. We also aimed to analyze whether transplantation of autologous ADSCs combined with ShakeGel™3D could improve the fertility of IUA mice.

## Materials and methods

### Experimental animals

All animal experimental protocols were approved by the Second Affiliated Hospital of Fujian Medical University Animal Care Committee and were in accordance with the National Institutes of Health Guide for the Care and Use of Laboratory Animals (number 97). Mice were kept in standard cages at 22 °C with a 12-h light-dark cycle and were allowed unlimited access to food and water.

### Autologous ADSC preparation and identification

Mouse autologous ADSCs were prepared and cultured as described in previous studies [31–33]. Briefly, inguinal adipose tissue was obtained from female C57BL/6 mice 3–4 weeks of age, and each mouse was given an ear tag with a certain number on it. Fat tissue was collected and enzymatically digested with 0.75 mg/mL collagenase type II (Sigma) for 30 min at 37 °C. Immediately after digestion, the sample was centrifuged, and the precipitate was retained. For culture, the precipitate was rinsed twice in PBS, and the cell pellet was re-suspended in DMEM/F12 (1:1) (Hyclone) containing 10% fetal bovine serum (Gibco), 1% pen-strep (Gibco) and cultured at 37 °C in a 5% CO_2_ humidified incubator. Flow cytometric analysis was used to identify cell surface markers of ADSCs, including CD105 (BioLegend), CD29 (BioLegend), CD75 (BioLegend), CD34 (BioLegend), and CD45 (BioLegend). Cells were treated with commercial osteogenic, chondrogenic, or adipogenic medium (Cyagen) for up to 24 days, following the manufacturer’s instructions. The ability of ADSCs to differentiate through adipogenesis, osteogenesis, and chondrogenesis was visualized by Oil Red O staining, Alizarin red staining, and Alcian blue staining (Cyagen), respectively. Autologous ADSCs from passage 3 were used for experiments.

### Preparation of the mouse autologous ADSCs combined with ShakeGel™3Dcomplex

The complex was made as follows: First, the ShakeGel™3D (Biomaterials USA) solution, which has been reported to improve cell culture strategies [20], was gently mixed with the cell suspension into a centrifuge tube. Second, cell culture wells were pre-wet with 1× sterile PBS, and then the gel-cell mixture was rapidly added to the well and the mix was gently rocked back and forth to spread the mixture evenly across the surface of the well. Third, incubation followed at 37 °C for 5–10 min for hydrogel formation. Fourth, culture medium was added along the plate wall, followed by incubation at 37 °C in a humidified atmosphere containing 5% CO_2_. Finally, after 24 h, half the volume of the old medium was discarded, and the same amount of fresh medium was added. After that, the medium was refreshed every 24 h. According to the manufacturer’s protocol, Calcein AM (Abcam) was used to examine cell viability, and cell counting Kit-8 (CCK-8) (Dojindo) was used to evaluate the proliferation of ADSCs. Three samples from each group were assessed using the CCK-8 assay (*n* = 3).

### Establishment of IUA model

**Fig. 1:**
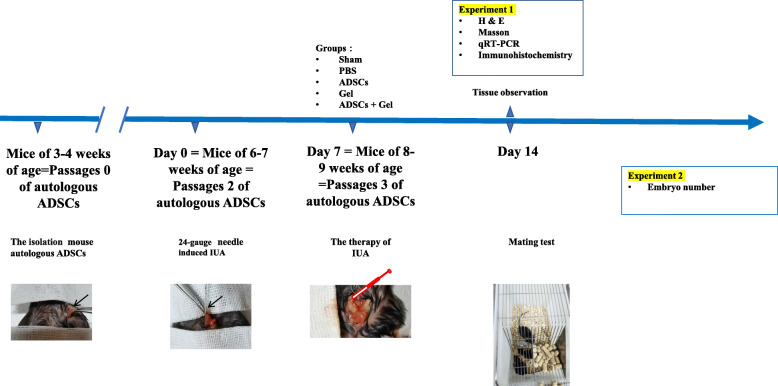
Study design

When the cells reached the second generation, which is approximately 6 or 7 weeks in a state of diestrus cycles of mice with consecutive 4-day estrus cycles [34], the IUA mouse model was established according to previous research [34]. Seven days after the surgery, the autologous ADSCs were cultured to the third generation. The mice (*n* = 50) were subdivided into 5 groups: sham operation group (sham group, *n* = 10), where mice had laparotomy without any treatment; PBS model group (PBS group, *n* = 10), where mice underwent induction of intrauterine adhesions, and, 7 days after modeling, a relaparotomy was performed and the mice were injected with 10 μl PBS in each of the two uterine horns; autologous ADSCs treatment group (ADSCs group, *n* = 10), where a relaparotomy was performed after 7 days of modeling, and autologous ADSCs (5*10^6^ suspended in 10 μl PBS) [35] were injected into each of the two uterine horns, one by one, according to the mouse’s ear tag; ShakeGel™3D model group (Gel group, *n* = 10), where mice underwent induction of intrauterine adhesions 7 days after modeling, a relaparotomy was performed, and the mice were injected with 10 μl ShakeGel™3D in each of the two uterine horns; and ADSCs combined with ShakeGel™3D treatment group (ADSCs + Gel group, n = 10), where a relaparotomy was performed after 7 days of modeling, and ADSCs combined with ShakeGel™3D (5*10^6 suspended in 10 μl ShakeGel™3D) were injected into each of the two uterine horns, one by one, according to the mouse’s ear tag. Seven days after treatment, 25 mice (*n* = 5 per group) were sacrificed, their uterine tissues were harvested for the next experiment, and the other 25 remained for mating experiments. The study design is shown in Fig. 1.

### GFP labeling of ADSCs

The green fluorescent protein (GFP) of a lentivirus (OBIO Technology) was stored at − 80 °C. According to the manufacturer’s protocol, after 72 h of virus infection, the cells were observed under a fluorescence microscope to determine the efficiency of cell infection. These cells were then injected into the uterus.

### Hematoxylin and eosin (H&E) staining

Uteri were fixed in 4% paraformaldehyde for 24 h and embedded in paraffin. The slides were first deparaffinized and rehydrated, and then stained with H&E (Solarbio). We visualized the whole image of the pathological uterus and counted the gland quantity to predict IUA. Image-pro plus 6.0 was used to calculate the number of glands and endometrial thickness, and 3–5 fields were randomly selected from each slide to determine the mean number of glands and mean endometrial thickness.

### Masson staining

Masson staining was used to detect endometrial fibrosis. The deparaffinized and rehydrated slides were incubated with Masson staining mixture (Solarbio) for 5 min and then stained with phosphomolybdic acid-aniline blue solution (Solarbio) for 5 min. The areas of the collagen fibers stained in blue relative to the total view were calculated.

### Quantitative real-time polymerase chain reaction (qRT-PCR)

Total RNA was extracted from macrophages using the Mini BEST Universal RNA Extraction Kit (TaKaRa). RNA purity and concentration were measured using a spectrophotometer. Reverse transcription and cDNA synthesis were performed using PrimeScript™ RT reagent Kit with gDNA Eraser (Perfect Real Time) (Takara, Japan) according to the manufacturer’s instructions. The mRNA expression of α-SMA, TGF-β1, Smad-5, and BMP7 was quantified by qRT-PCR with TB Green® Premix Ex Taq™ (TliRNaseH Plus) (Takara), and the housekeeping gene, glyceraldehyde 3-phosphate dehydrogenase (GAPDH), was used as an internal control for mRNA abundance. The sequences of the forward and reverse primers are shown in Table 1. Each group included six samples, and each sample was performed in triplicate. Relative gene expression between groups was calculated using the 2^-△△CT^ method.

**Table 1:** qRT-PCR Forward/Reverse(F/R) primers sequences

Primers	Sequences 5′-3′
**a-SMA**	F: 5′- GCGTGGCTATTCCTTCGTGACTAC -3′
	R: 5′- CGTCAGGCAGTTCGTAGCTCTTC -3′
**TGF-β1**	F: 5′- CCAGATCCTGTCCAAACTAAGG -3′
	R: 5′- CTCTTTAGCATAGTAGTCCGCT -3′
**BMP7**	F: 5′- GATCCTGTCCATCTTAGGGTTG -3′
	R: 5′- GTTGTACAGGTCCAACATGAAC -3′
**Smad5**	F: 5′- TTTCCCCTTATCTCCTAACAGC -3′
	R: 5′- TACTGCTTGTATCCATAGGCTG -3′
GAPDH	F: 5′- TGGGTGTGAACCATGAGAAG-3′
	R: 5′- GCTAAGCAGTTGGTGGTGC-3′

*a-SMA* a-smooth muscle actin, *TGF-β1* transforming growth factor beta receptor I, *BMP7* bone morphogenetic protein 7, *Smad5* SMAD family member 5, *GAPDH* glyceraldehyde-3-phosphate dehydrogenase

### Immunohistochemistry

After deparaffinization, dehydration, and antigen retrieval, the slides were immersed in 3% hydrogen peroxide to block endogenous peroxidase activity, and then they were blocked in goat serum for 1 h. The slides were then incubated overnight at 4 °C with primary antibodies against α-smooth muscle actin (α-SMA, Abcam, 1:2000), TGF-β1 (Abcam,1:500), BMP7 (Abcam, 1 μg/ml), and Smad5 (Abcam, 1:800). The sections were then incubated with appropriate secondary antibodies for 60 min at room temperature. The slides were then stained with 3, 3-diaminobenzidine (DAB) at room temperature, lightly counterstained with hematoxylin, dehydrated, and covered with glass cover slips. Quantification of immunoreactivity was performed using Image Pro-Plus 6.0, and 3–5 fields were randomly selected from each slide to determine the mean optical density (MOD).

### Statistical analysis

Data were analyzed using the *F* test with subsequent t-tests (equal variance) for the comparison between two different groups. For three or more groups, an ANOVA test was used, followed by the least significant differences method. GraphPadPrism8 (GraphPad Software Inc., La Jolla, CA, USA) was used to obtain graphs and statistics. Significant values were designated as follows: *p < 0.05, **p < 0.01, ***p < 0.001, and ****p < 0.0001. All data are shown as the mean ± standard deviation (SD).

## Results

### Characterization and differentiation of autologous ADSCs and their safety assessment on ShakeGel™3D

The appearance of autologous ADSCs from passage 0 (P0) to P3, especially the third passage, gradually came to resemble typical spindle-shaped fibroblast-like cells that were arranged closely with vortex-like growth (Supplementary figure S1.1). In the third passage, fluorescence-activated cell sorting (FACS) showed that autologous ADSCs expressed CD105 (99.1%), CD29 (99.6%), CD73 (98.9%), CD34 (0.46%), and CD45 (3.26%) (Supplementary figure S1.2). In addition, these cells were successfully induced to become adipocytes, osteoblasts, and chondroblasts in vitro (Supplementary figure S1.3).

**Fig. 2:**
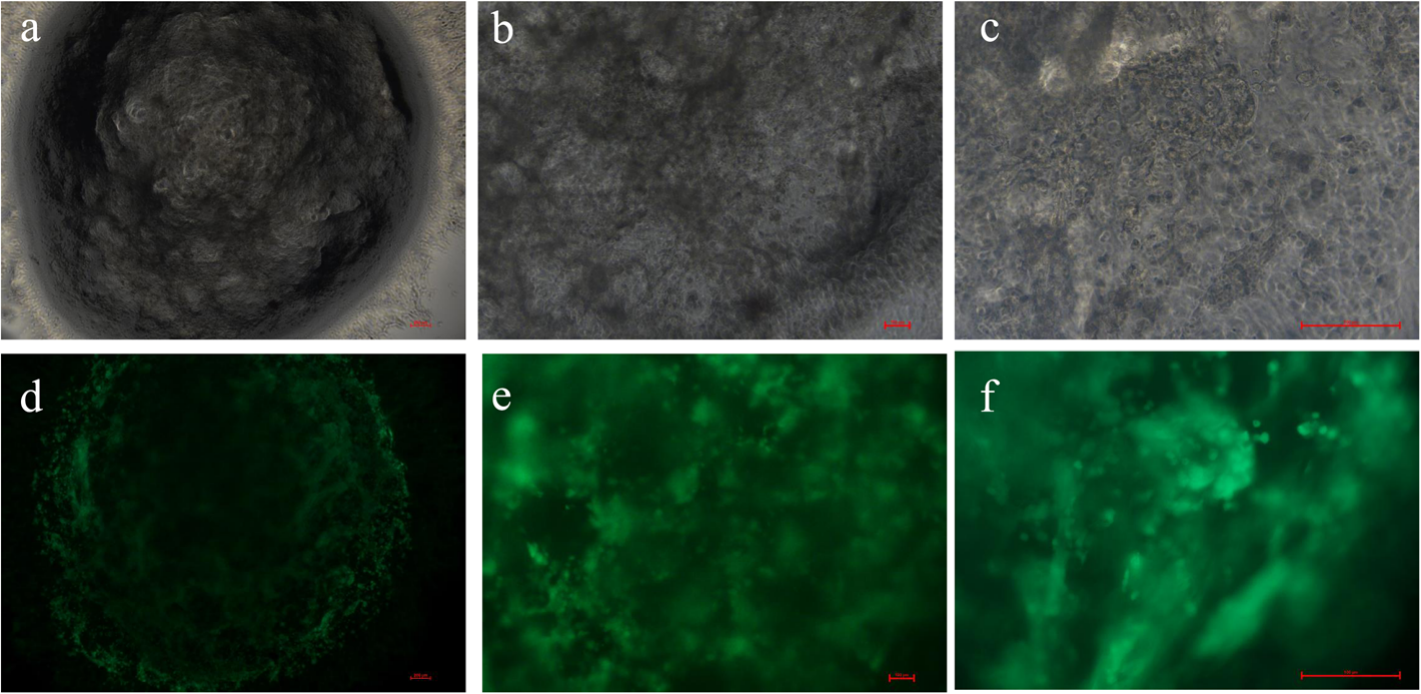
Assay of the activity of ADSCs combined with ShakeGel™3D. **a**, **b**, and **c** respectively were ADSCs combined with ShakeGel™3D under white light 4×, 10×, and 20**×**. **d**, **e**, and f respectively were ADSCs combined with ShakeGel™3D under active staining 4×, 10×, and 20× (scale bar = 100 μm)

**Fig. 3:**
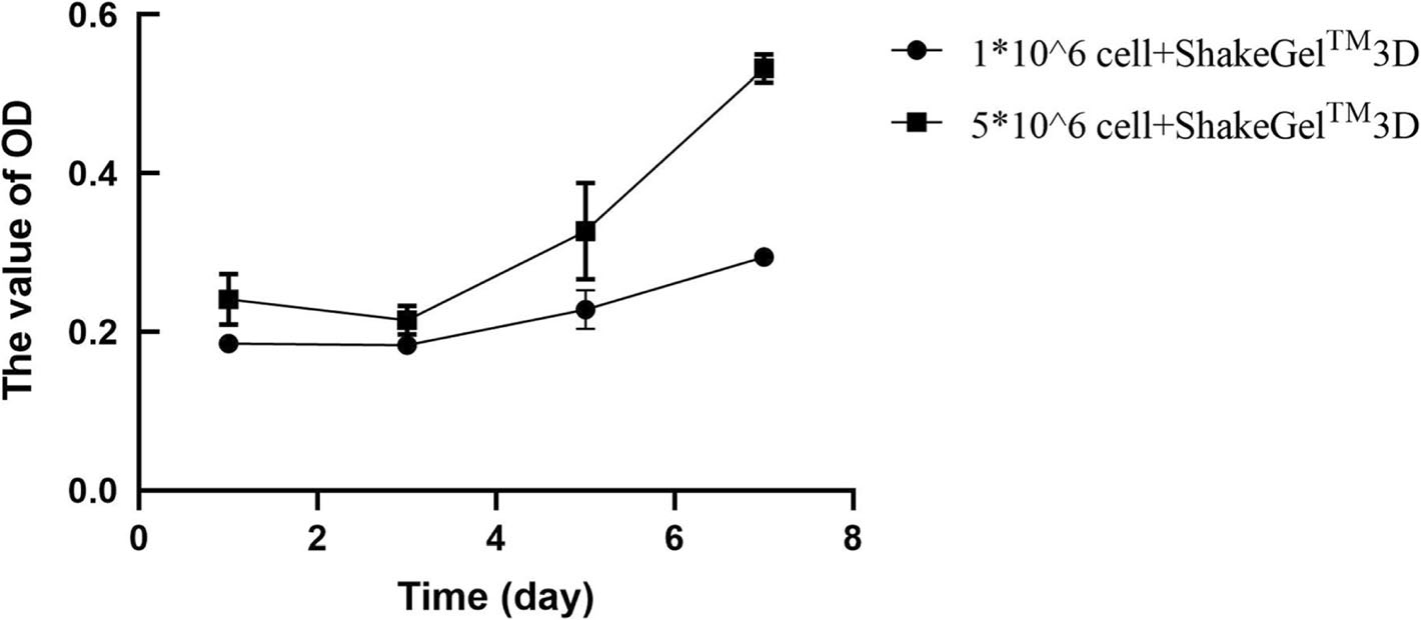
The results of CCK-8 in 1*10^6^ ADSCs and 5*10^6^ ADSCs co-culture with ShakeGel™3D

To further evaluate the safety of autologous ADSCs on ShakeGel™3D, the live-dead cell staining results (Fig. 2) showed that these cells showed aggregation of globulin in the ShakeGel™3D, and a small number of fibrous fat stem cells were extended at the junction between the ShakeGel™3D edge and the cell culture base. On day seven in the ShakeGel™3D culture environment, autologous ADSCs showed good activity and could continue to proliferation in the shaker environment. In addition, the CCK-8 results (Fig. 3) verified that the OD value of cells cultured by 5*10^6^ cells + ShakeGel™3D was higher than that by 1*10^6^ cells + ShakeGel™3D.On the third day of co-culture, the OD value began to increase gradually. By the seventh day, the OD value of 5*10^6^ cells + ShakeGel™3D was over 50%.

### The IUA models the effect of autologous ADSCs on ShakeGel™3D

**Fig. 4:**
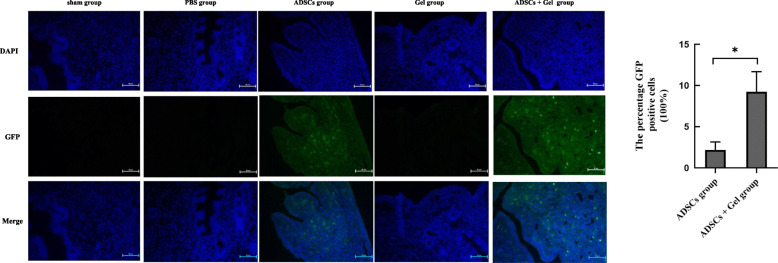
Engraftment of the endometrium with ADSCs following local injection. **4.1** Fluorescence microscopy analysis of uterine tissue sections (scale bar = 50 μm). **4.2** The percentage GFP-positive cells of ADSCs + Gel group (9.215 ± 2.470%, *n* = 3) was higher than ADSCs group (2.175 ± 0.9816%, *n* = 3) (*P<0.05)

First, to locate the autologous ADSCs/GFP in the uterus, as expected, we did not observe any GFP-positive cells in the uterine tissue sections of the sham, PBS, and Gel groups. However, we observed GFP-positive cells in the uterine tissue sections of the ADSCs group (2.175%) and ADSCs + Gel group (9.215%). Furthermore, GFP-positive cells in the uterine tissue sections of the ADSCs + Gel group were higher than that in the ADSCs group (*P* = 0.0101) (Fig. 4).

The macroscopic appearance of the uterus sham groups was smoother than in the PBS group. On day 7 from ADSCs transplantations, ShakeGel™3D, or ADSCs combined with ShakeGel™3D, the macroscopic appearance of the uterus gradually recovered, similar to that of a normal uterus (Fig. 5.1). H&E staining of the uterine tissue showed that the endometrial thickness (163.8 ± 6.128 μm) of the PBS group showed significant differences when compared with the thickness (264.5 ± 31.75 μm) of the sham group (*P* < 0.0001) (Fig. 5.2). When transplanted with ADSCs or ADSCs combined with ShakeGel™3D, thickness increased significantly compared to the PBS group (ADSCs group 190.1 ± 8.389 μm, *P* < 0.05; ADSCs + Gel group 228.8 ± 2.254 μm, *P* < 0.0001). The endometrium regenerated in the ADSCs + Gel group was thicker than that in the ADSCs group (*P* < 0.001) (Fig. 5.2). Further, when comparing to endometrial tissues of the PBS group, gland numbers were increased in the ADSCs group (9.182 ± 2.857 versus 1.545 ± 0.9342, respectively, per unit area, *P* < 0.0001), Gel group (14.55 ± 1.293 versus 1.545 ± 0.9342, respectively, per unit area, *P* < 0.0001), and ADSCs + Gel group (19.36 ± 2.063 versus 1.545 ± 0.9342, respectively, per unit area, P < 0.0001). Compared with the ADSCs group, the gland numbers of ADSCs +Gel were increased (P < 0.0001). To further evaluate the degree of fibrosis, Masson’s trichrome staining was performed. As shown in Fig. 5.3, a large amount of fibrous tissue appeared in the PBS group (56.89 ± 0.4425%), compared to the sham group (29.44 ± 5.2305%). Interestingly, the percentage of fibrosis area was decreased after ADSCs (50.10 ± 1.280%, *P* < 0.05), ShakeGel™3D (45.17 ± 1.196%, *P* < 0.0001) or ADSCs combined with ShakeGel™3D (39.55 ± 0.4125%, *P* < 0.0001) treatment compared with the PBS group. In addition, the percentage of fibrosis area in the ADSCs +Gel group was significantly less than that of the ADSCs group (*P* < 0.001).

**Fig. 5:**
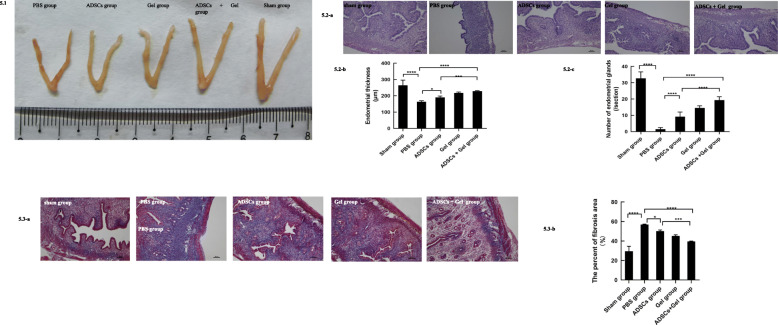
Uterine morphological features and changes. **5.1** The macroscopic appearance of the uterus. **5.2-a** H&E staining of mice uterine tissue (scale bar = 50 μm). **5.2-b** The endometrial thickness at transplantation day seven. Sham group, 264.5 ± 31.75 μm; PBS group, 163.8 ± 6.128 μm; ADSCs group, 190.1 ± 8.389 μm; Gel group, 217.8 ± 5.839 μm; ADSCs + Gel, 228.8 ± 2.254 μm. **5.2-c** The number of endometrial glands at transplantation day seven. Sham group, 32.73 ± 3.952/section; PBS group, 1.545 ± 0.9342/section; ADSCs group, 9.182 ± 2.857/section; Gel group, 14.55 ± 1.293/section; ADSCs + Gel, 19.36 ± 2.063/section. *P < 0.05, **P < 0.01,***P < 0.001, ****P < 0.0001. **5.3-a** Masson staining of mice uterine tissue (Scale bar = 50 μm). **5.3-b** The percent of fibrosis area at transplantation day seven. Sham group, 29.44 ± 5.2305%; PBS group, 56.89 ± 0.4425%; ADSCs group, 50.10 ± 1.280%; Gel group, 45.17 ± 1.196%; ADSCs + Gel, 39.55 ± 0.4125%. *P < 0.05, **P < 0.01, ***P < 0.001, ****P < 0.0001, ns *P* < 0.05

**Fig. 6:**
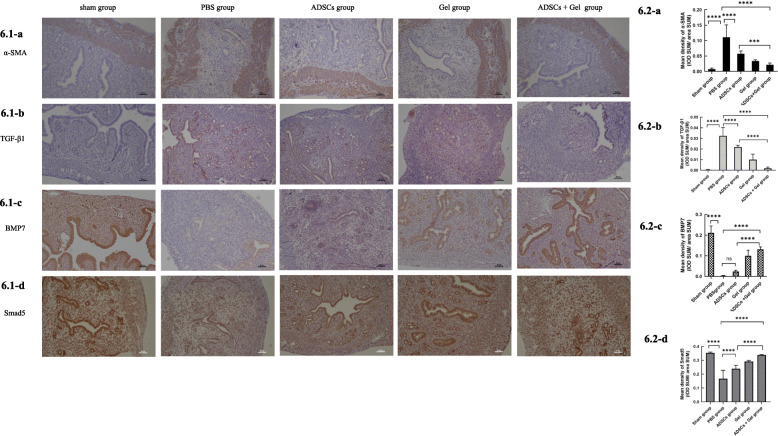
Immunohistochemistry results **6.1** Representative immunostaining of α-SMA, TGF-β1, BMP7, and Smad5 (scale bar = 50 μm). **6.2-a** Mean density of α-SMA, Sham group, 0.007813 ± 0.003258; PBS group, 0.1109 ± 0.04010; ADSCs group, 0.05746 ± 0.008630; Gel group, 0.03392 ± 0.003734; ADSCs + Gel, 0.02151 ± 0.005611. **6.2-b** Mean density of TGF-β1, Sham group, 0.0004134 ± 0.0004932; PBS group, 0.03246 ± 0.007779; ADSCs group, 0.02180 ± 0.001778; Gel group, 0.009999 ± 0.005183; ADSCs + Gel, 0.001736 ± 0.001229. **6.2-c** Sham group, 0.2119 ± 0.03328; PBS group, 0.002817 ± 0.002115; ADSCs group, 0.02330 ± 0.006284; Gel group, 0.09967 ± 0.02718; ADSCs + Gel, 0.1314 ± 0.01210. **6.2-d** Mean density of Smad5, Sham group, 0.3536 ± 0.005790; PBS group, 0.1676 ± 0.06002; ADSCs group, 0.2391 ± 0.02442; Gel group, 0.2910 ± 0.009053; ADSCs + Gel, 0.3372 ± 0.004266. *P < 0.05, **P < 0.01, ***P < 0.001, ****P < 0.0001, ns *P* >0.05

To further confirm the results of the fibrosis of experiments, we detected α-SMA expression and mean optical density of α-SMA in endometrium (Fig. 6). All these valuations indicated that the fibrosis in the ADSCs + Gel group was significantly smaller than in the ADSCs group. In addition, expression of TGF-β1, an archetypical promoter of fibrosis, (Fig. 6.2-b) was significantly less in the ADSCs + Gel group than in the ADSCs group, as shown by qRT-PCR (Fig. 7.1-b) and IHC experiments. The mating experiments showed that only the Sham group and ADSCs + Gel group achieved pregnancy (Fig. 7.2).

**Fig. 7:**
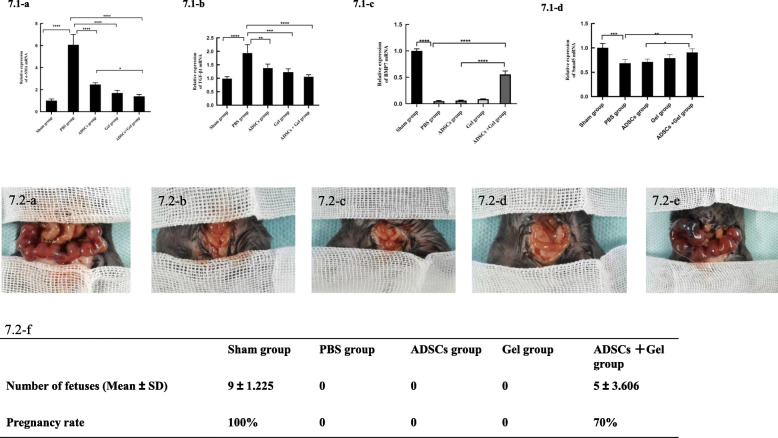
**7.1-a** Relative expression of α-SMA mRNA. Sham group, 1.008 ± 0.1544; PBS group, 0.1544 ± 0.9503; ADSCs group, 2.474 ± 0.1598; Gel group, 1.703 ± 0.2415; ADSCs + Gel, 1.398 ± 0.1797. **7.1-b** Relative expression of TGF-β1 mRNA. Sham group, 1.001 ± 0.05932; PBS group, 1.951 ± 0.2980; ADSCs group,1.391 ± 0.1269; Gel group, 1.227 ± 0.1269; ADSCs + Gel, 1.068 ± 0.06639. **7.1-c** Relative expression of BMP7 mRNA. Sham group, 1.001 ± 0.03872; PBS group, 0.05062 ± 0.01281; ADSCs group, 0.06124 ± 0.006388; Gel group, 0.08836 ± 0.005922; ADSCs + Gel, 0.5575 ± 0.06289. **7.1-d** Relative expression of Smad5 mRNA. Sham group, 1.003 ± 0.08697; PBS group, 0.6843 ± 0.07691; ADSCs group, 0.7127 ± 0.05734; Gel group, 0.7891 ± 0.07409; ADSCs + Gel, 0.9066 ± 0.07593. *P < 0.05, **P < 0.01, ***P < 0.001, ****P < 0.0001, ns *P* >0.05. **7.2** Results of the mating experiment. Reproductive outcomes in the sham group (**7.2-a**), PBS group (**7.2-b**), ADSCs group (**7.2-c**), Gel group (**7.2-d**), and ADSCs + Gel group (**7.2-e**). The number of fetuses in each group was analyzed (**7.2-f**). Data are presented as mean ± SD. The pregnancy rate is equal to the number of pregnant uteri divided by the total number of uteri

### ADSCs combined with ShakeGel™3D treatment enhance BMP7-Smad5 signaling

The qRT-PCR results (Fig. 7.1) showed that BMP7 expression in the ADSCs group (0.06124 ± 0.006388) was slightly higher than in the PBS group (0.05062 ± 0.01281), but the difference was not statistically significant (*P* = 0.5302). BMP7 expression was higher in ADSCs + Gel group (0.5575 ± 0.06289) than in the PBS group. The difference was statistically significant (*P* < 0.0001). The ADSCs + Gel group was also statistically significant compared to the ADSCs group (P < 0.0001). Similarly, Smad5 expression in the ADSCs group (0.7127 ± 0.05734) was slightly higher than in the PBS group (0.6843 ± 0.07691), but the difference was not statistically significant (*P* = 0.9820). The mRNA expression of Smad5 in the ADSCs + Gel group (0.9066 ± 0.07593) was significantly higher than in the PBS group (*P* < 0.01). The ADSCs + Gel group result was also statistically significant compared with ADSCs (*P* = 0.0167).

Likewise, for immunohistochemistry results, the staining intensity for BMP7 in the ADSCs group (0.02330 ± 0.006284) was slightly higher than that in the PBS group (0.002817 ± 0.002115), but the difference was not statistically significant (*P* = 0.1739). BMP7 expression was higher in the ADSCs + Gel group (0.1314 ± 0.01210) than in the PBS group. The difference was statistically significant (*P* < 0.0001). Compared with the ADSCs group, the ADSCs + Gel group expression was significantly higher (*P* < 0.0001). The mean density of Smad5 in the PBS group (0.1676 ± 0.06002) was lower than in the ADSCs group (0.2391 ± 0.02442, *P* < 0.0001), Gel group (0.2910 ± 0.009053, *P* < 0.0001), and ADSCs + Gel group (0.3372 ± 0.004266, *P* < 0.0001). The mean density of Smad5 in the ADSCs + Gel group was significantly higher than in the ADSCs group (*P* < 0.0001) and Gel group (*P* = 0.0087).

## Discussion

In this study, we have demonstrated that (1) the combination of ShakeGel™3D and autologous ADSCs can achieve good results, thereby creating a feasible three-dimensional growth environment; (2) autologous ADSCs combined with ShakeGel™3D can promote the recovery of injured endometrial tissue, thus increasing the thickness of the endometrium, increasing the number of glands and reducing fibrosis; and (3) compared with other treatment groups, the combination of autologous ADSCs and ShakeGel™3D grafts can increase the expression of BMP7 and Smad5, indicating that the combination of autologous ADSCs and ShakeGel™3D grafts can promote endometrial repair through the BMP7-Smad5 signaling pathway.

Stem cells, including MSCs, are one of the most promising tools, and they have been applied to various tissues for regenerative medicine and cell therapy, including the uterus endometrium regeneration [35, 36]. ADSCs can be isolated from white adipose tissue in large quantities, and autologous transplantation can be realized. Some studies have shown that the application of allogeneic stem cell therapy has some risks, including abnormal immune reconstitution, secondary cancer, and graft-versus-host disease [3, 37, 38]. Our study is the first to use autologous ADSCs to treat intrauterine adhesions in mice. In our study, we used autologous ADSCs combined withShakeGel™3D, which has proven that it can support the microenvironment and tissue microstructure for cells to improve proliferation [20]. The CCK-8 results of our study showed that autologous ADSCs still proliferate in ShakeGel™3D. Furthermore, the effects of the group with autologous ADSCs combined with ShakeGel™3D were confirmed by histological inspection and detection of some fiber correlation factors. In addition, compared with the ADSCs and the Gel groups, the ADSCs + Gel group had more GFP-positive cells in the endometrial tissue, thickened endometrium, increased endometrial glands, decreased fibrotic area, and α-SMA and TGF-β1 expression. These results suggest that the ShakeGel™3D not only acts as a physical barrier to IUA, but also provides an ideal attachment site for ADSCs to maintain a high density in the uterine cavity, thereby improving reconstruction of abnormal tissue. Further, GFP positive cells were observed in the stromal compartment, consistent with a previous report [39]. We suggested that ADSCs may provide paracrine support for the epithelial cells to regenerate. Transplanted ADSCs may release bioactive molecules, including growth factors, cytokines, chemokines, and stem cell mobilizing factors that are beneficial to the IUA by paracrine mechanisms. Although Liu et al. concluded that systemic administration can recruit MSCs to the injured uterus better than local injection [35], vein engraftment of stem cells in the damaged area cannot be guaranteed. In fact, the majority of the stem cells after intravenous injection are trapped in the lungs, and the number of MSCs that home to the endometrium is very small [35, 39]. In addition, in our research, ShakeGel™3D serves as a slow-release stem cell scaffold, allowing ADSCs to continuously secrete factors to promote endometrial repair. Therefore, we used local injection of autologous ADSCs combined with ShakeGel™3D to treat IUA.

Interestingly, our study also showed that the Gel group without any therapeutic cells showed better performance (thicker endometrium, more glands, less fibrosis area after treatment) than the ADSCs group. We suggested that ShakeGel™3D acted as a physical barrier to IUA, retarded adverse remodeling processes, and provided an ideal extracellular environment for the remaining endometrial stromal cells. To figure out why ShakeGel™3D solely helps the endometrium regeneration, more researches need to be done. In addition, all kinds of hydrogel, as a stand-alone therapy, have been used in IUA human [40], which cannot completely eliminate IUA, but can attenuate the deterioration of adhesions. Further, many researchers have combined hydrogel biomaterials with stem cells, growth factors, and other components to treat diseases.

Abnormal repair and fibrosis after endometrial surgery can lead to intrauterine adhesion damage, which is pathologically similar to all fibrotic lesions, including the extracellular matrix, excessive accumulation of tissue remodeling, and scar formation [41]. Therefore, for IUA therapy, blocking the formation of fibrosis plays a key role. TGF-β1 has been shown to promote endometrial fibrosis in IUA patients and animal models [42]. Some researchers have proved that TGF-β1 influences tissue fibrosis via Smad2, Smad3, Smad7 signaling [43]. However, a previous study showed that the expression of BMP7, another member of the TGF-β superfamily, was negatively correlated with Smad5 in IUA. BMP7-Smad5 signal transduction occurs in endometrial fibrosis and has an anti-fibrotic effect in IUA [25]. Our study used mechanical trauma to establish a mouse model of IUA. We found that, compared with the PBS group, both the mRNA and protein BMP7 levels in the ADSCs + Gel group increased. At the same time, the level of Smad5 consistent with that of BMP7 also increased. Compared with the PBS group, the levels of BMP7 and Smad5 in the ADSCs group increased slightly, but the difference was no statistically significant. Our study shows for the first time that the combination of ADSCs and ShakeGel™3D can treat IUA by changing the BMP7-Smad5 signaling pathway. Furthermore, we suggest that the gel can improve the function of ADSCs.

We outline next few steps in our ongoing research to further elucidate the mechanism of ADSCs combined with gel in the treatment of intrauterine adhesions. First, due to the extreme challenge of preparing an appropriate number of autologous ADSCs, we plan to isolate and purify exosomes from ADSCs in place of cell therapy for IUA. In addition, we may be able to identify key factors in the release of ADSCs during endometrial recovery. Second, we have just completed a mouse study, and we are planning to treat IUA with autologous ADSCs combined with ShakeGel™3D in a large animal model more similar to the pathophysiology of the endometrium of human females. In addition, randomized controlled trials are needed to confirm the therapeutic role of ADSCs in fertility medicine.

## Conclusion

In this study, it was shown that both autologous ADSCs and autologous ADSCs combined with ShakeGel™3D could repair IUA caused by mechanical injury. Since ShakeGel™3D enhances the function of ADSCs, autologous ADSC transplantation combined with ShakeGel™3D holds significant advantages in anti-fibrotic repair and promotion of endometrial regeneration by altering the BMP7-Smad5 signaling pathway. These data are helpful in treating intrauterine adhesions.

## Supplementary Information

**Additional file 1: Supplementary figure S1. S1.1** The appearance of autologous ADSCs of passage 0 (P0) to P3. The cells at third passage showed homogenous fibroblastic morphology. **S1.2** Adipose tissue derived mesenchymal stem cell identification with flow cytometry. CD105: 99.1%, CD29: 99.6%, CD73: 98.9%, CD34: 0.46%, CD45: 3.26%. **S.3** The differentiation of ADSCs. Under the white light, The differentiated adipocytes secreted lipid droplets (a-1, a-2). Oil Red-O staining adipogenic differentiation (b-1, b-2). Alizarin red staining indicated osteogenic differentiation (c-1, c-2). Alcian blue staining indicated chondrogenic differentiation (d-1, d-2).

## Data Availability

The data that support the findings of this study are available from the corresponding author upon reasonable request.

## Abbreviations

IUA: Intrauterine adhesions
MSCs: Mesenchymal stem cells
ADSCs: Adipose-derived stem cells
BMP7: Bone morphogenetic protein 7
TGF: Transforming growth factor
