# Supplementary figures and images for: Repair abilities of mouse autologous adipose-derived stem cells and ShakeGel™3D complex local injection with intrauterine adhesion by BMP7-Smad5 signaling pathway activation
Source: Stem Cell Res Ther. 2021 Mar 18;12:191. doi: 10.1186/s13287-021-02258-0 (PMC7977602; doi:10.1186/s13287-021-02258-0)

## Slide 1
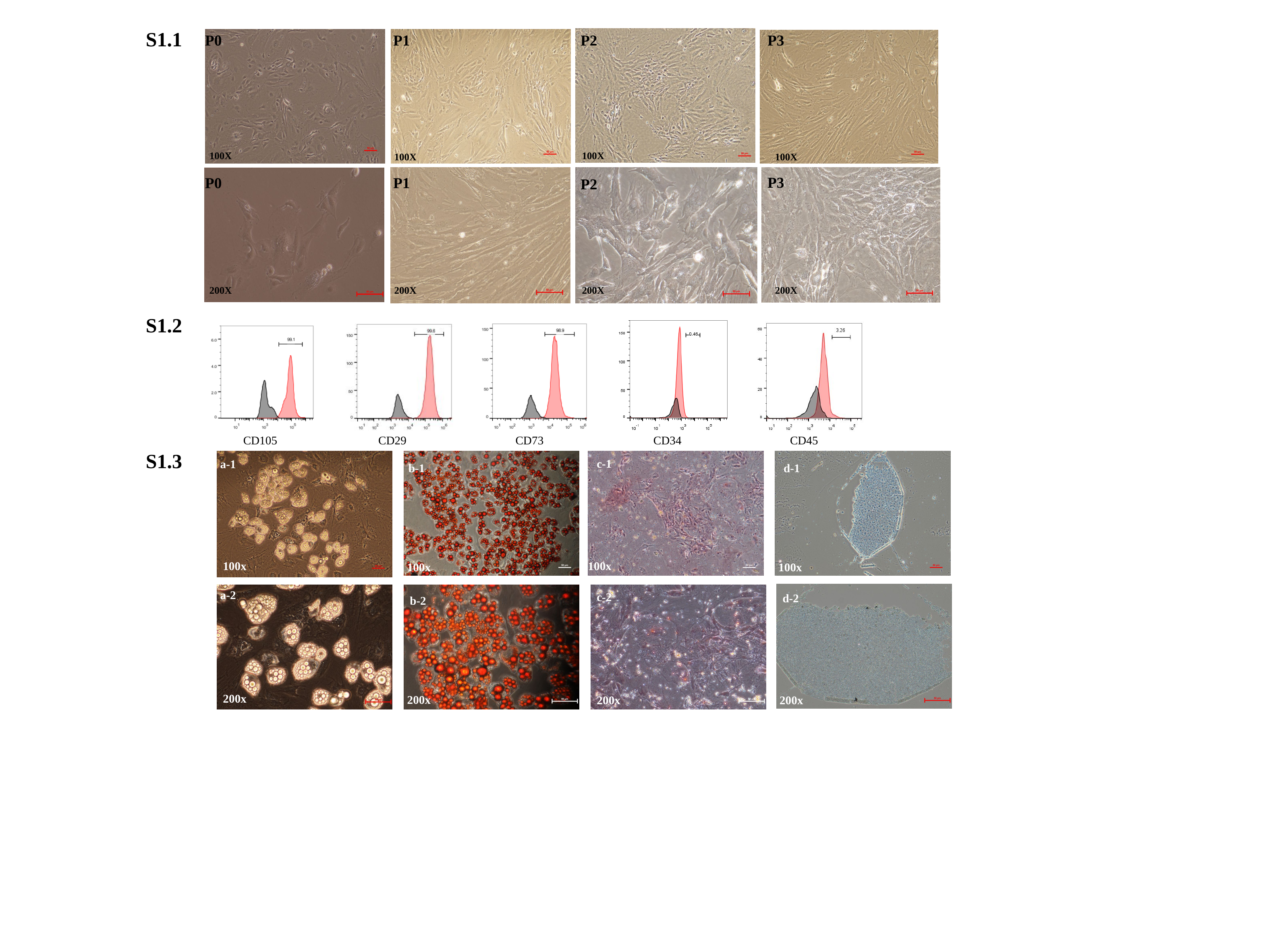

S1.1
P0
P1
P2
P3
100X
100X
100X
100X
P3
P1
P0
P2
P1
200X
200X
200X
200X
S1.2
CD105
CD29
CD73
CD34
CD45
S1.3
c-1
a-1
d-1
b-1
100x
100x
100x
100x
a-2
c-2
d-2
b-2
200x
200x
200x
200x

Supplement: Supplementary file 1 — Additional file 1: Supplementary figure S1. S1.1 The appearance of autologous ADSCs of passage 0 (P0) to P3. The cells at third passage showed homogenous fibroblastic morphology. S1.2 Adipose tissue derived mesenchymal stem cell identification with flow cytometry. CD105: 99.1%, CD29: 99.6%, CD73: 98.9%, CD34: 0.46%, CD45: 3.26%. S.3 The differentiation of ADSCs. Under the white light, The differentiated adipocytes secreted lipid droplets (a-1, a-2). Oil Red-O staining adipogenic differentiation (b-1, b-2). Alizarin red staining indicated osteogenic differentiation (c-1, c-2). Alcian blue staining indicated chondrogenic differentiation (d-1, d-2). [file 13287_2021_2258_MOESM1_ESM.pptx]
